# miR-21 Regulates Immune Balance Mediated by Th17/Treg in Peripheral Blood of Septic Rats during the Early Phase through Apoptosis Pathway

**DOI:** 10.1155/2022/9948229

**Published:** 2022-04-27

**Authors:** Cheng Liu, Qi Zou

**Affiliations:** Department of Critical Care Medicine, The First Affiliated Hospital of Bengbu Medical College, Anhui, Bengbu 233004, China

## Abstract

**Objective:**

To study the mechanism by which miR-21 regulates the differentiation and function of Th17/Treg cells in sepsis.

**Methods:**

A rat model with sepsis was made by cecal ligation and puncture (CLP). Then, some of the septic rats were transfected with miR-21 mimic or inhibitor by liposome. At 48 hours, lymphocytes and plasma from septic rats were isolated for further experimental detection. The expression of miR-21 in lymphocytes was detected by Polymerase Chain Reaction (PCR); the differentiation of Th17/Treg cells was counted by flow cytometry; lymphocyte apoptosis was observed by terminal deoxynucleotidyl transferase-mediated dUTP-biotin nick end labeling (TUNEL) assay. The caspase-3/9 proteins were tested by Western blot; IL-10 and IL-17 were detected by enzyme-linked immunosorbent assay (ELISA).

**Results:**

Compared with the sepsis group (SP group), the Th17 cells increased significantly, the Treg cells decreased significantly, the apoptosis rate of lymphocytes decreased significantly, the mRNA and proteins of caspase-3/9 decreased significantly, the IL-17 decreased, and the IL-10 increased in the sepsis group transfected with miR-21 (SP + miR-21 mimic group). After transfection of miR-21 inhibitor, the results were almost opposite to those of SP + miR-21 mimic group.

**Conclusions:**

The differentiation and function of Th17/Treg cells were regulated by miR-21 in sepsis through caspase pathway.

## 1. Introduction

At present, sepsis is still one of the major diseases affecting the safety of human life in the world. In 2017, it was reported that 49 million cases of sepsis were recorded in the world, with 11 million deaths. This study pointed out that sepsis was one of the diseases with heavy medical burden in East Asia, Southeast Asia, South Asia, and South Africa [1]. Although the mortality rate of sepsis is very high, there is still no specific drugs that can effectively improve the prognosis [2]. It is widely known that “excessive inflammatory response” and “immune paralysis” are critical aspects in the pathophysiology of sepsis. Lymphocytes are very important in immune and inflammatory responses, especially Th17/Treg cells. Studies in different fields have confirmed that Th17/Treg cells are closely related to inflammatory response. For example, Yan et al. have found that the imbalance between Th17 and Treg cells was one of the causes of inflammatory enteritis. Th17 promoted tissue inflammation, while Treg inhibited tissue immune response [3]. Moaaz et al. have also found that the imbalance of Th17/Treg in autistic children led to the increase of proinflammatory factors and the decrease of anti-inflammatory factors [4]. Some scholars have proposed that the imbalance of Th17/Treg cells led to the disorder of inflammatory response, which promoted the occurrence and development of chronic obstructive pulmonary disease [5]. Similarly, one study has shown that overexpression of TGF-*β*1 could affect the level of inflammation in the lung tissue of septic mice by regulating the imbalance of Th17/Treg cells [6]. Another study also found that maresin-1 regulated the disorder of Th17/Treg by increasing the number of Treg cells and reducing the number of Th17 cells in the early stage of sepsis, so as to significantly inhibit the excessive inflammatory response in the process of acute lung injury and improve lung function [7]. With the development of the relevant research, the Th17 and Treg cells have been gradually elaborated in sepsis [8, 9]. The Th17/Treg ratio can change immune function and lead to the occurrence and development of sepsis [9, 10]. Therefore, regulating the Th17/Treg ratio may be one of the effective therapeutic strategies of sepsis.

Many studies have pointed out that apoptosis of lymphocyte can lead to the impairment of immune cells in sepsis, such as T lymphocytes, B lymphocytes, and NK cells [11]. Meanwhile, other studies have shown that improving the apoptosis of immune cells can improve the survival rate in sepsis [12–14]. So, it is of great significance to regulate the Th17/Treg cells ratio and promote the immune balance by preventing the apoptosis of lymphocytes in sepsis.

The researches have shown that microRNAs can regulate the differentiation and apoptosis of many cells [15, 16] and are very important in controlling the development and function of immune cells [17]. miR-21 is one of the most studied members of microRNAs family. Early studies have shown that miR-21 has a positive regulatory effect on Treg cells in Kawasaki disease [18], thus affecting the Th17/Treg ratio. Whether miR-21 can regulate the Th17/Treg ratio and affect the apoptosis of lymphocytes in sepsis is still unclear. Therefore, in our study, miR-21 was supposed to regulate the apoptosis of lymphocytes in sepsis, then changed the function and differentiation of Th17/Treg cells, and ultimately improved the immune and inflammation status of sepsis.

## 2. Materials and Methods

### 2.1. Grouping of Septic Animal Models

The animal experiment has been approved by the Ethics Committee of Bengbu Medical College (Ethics Statement Approval No.: 2018074). The model of cecal ligation and perforation was established in rats (CLP model); SD rats (male) weighing 200–250 g were selected; 5% Pentobarbital Sodium (5 mg/100 g) was injected intraperitoneally for anesthesia, and we found no signs of peritonitis, pain, or discomfort. After disinfection of the abdominal operation area, we cut the abdominal cavity about 2 cm along the abdominal white line to find out the cecum, ligated about one-third of the cecum with suture, punctured the ligated cecum twice with 21 g needle, and put the cecal ileum into the abdominal cavity and sutured. Finally, the rats were placed in the heating pad to maintain the anal temperature at 37 ± 0.5°C. The rats were divided into four groups by transfection in vivo, with eight rats in each group: control (NC rat) group, septic rat (SP rat) group, septic rats injected with miR-21 mimic (SP rat + miR-21 mimic) group, and septic rats injected with miR-21 inhibitor (SP rat + miR-21 inhibitor) group. The lymphocytes were also divided into four groups by transfection in vitro: NC group, SP group, SP + miR-21 mimic group, and SP + miR-21 inhibitor group. The rats were euthanized by cervical dislocation at the end of the study after the animals were anesthetized. We observed whether the breathing and heartbeat of the rats were completely stopped to confirm whether the rats died. The weight of the rats at the time of death was basically the same as that before the experiment (weighing 200–250 g). Based on the humane endpoints of the experimental animal center, we observed the animal performance every day during the experiment; if the rats were excessively listless or their body weight decreased >20%, we would terminate the experiment of the animals.

### 2.2. Isolation and Culture of Lymphocytes

Lymphocytes in peripheral blood of rats were isolated by density gradient method. We used heart puncture blood collection method to collect the blood sample (2 ml) after the animals were anesthetized. After adding the lymphocyte isolation solution (USA, sigma) into the centrifuge tube, we carefully add the diluted peripheral blood (peripheral blood : dilution = 1 : 2), 1500 r/min; after centrifugation for 30 min, the lymphocytes above the isolation solution were obtained for subsequent culture. The lymphocytes were cultured in vitro with serum and RPMI-1640 medium.

### 2.3. Cell Transfection

The mixing ratio (1 : 1–1 : 2/liposome volume: miR-21 mimic/inhibitor) was selected to transfect cells. The transfected liposome was Lipofectamine 3000 (lipo3000, Invitrogen L3000015). A suitable volume of serum-free medium and a suitable volume of transfection reagent were added to a transfection tube and cultured in incubator at 37°C for 48 h.

### 2.4. Apoptotic Lymphocyte Was Tested by Flow Cytometry

Lymphocytes were collected and labeled with CD4-FITC (eBioscience 11-0040-82, USA), CD25 (eBioscience 12-0390-82, USA), IL-17-PE (12-7177-81, eBioscience, USA), and FOXP3 (35-5773-82, eBioscience, USA). The phenotype of cells was tested by flow cytometry (FACSCalibur, Becton-Dickinson, USA).

### 2.5. The mRNAs of miR-21 and Caspase-3/9 Were Detected by PCR

Add Trizol reagent lysate and chloroform (5 : 1) to the separated lymphocyte sample, shake, and centrifuge. Add 75% ethanol to the sample of Trizol reagent lysate, mix well, centrifuge to precipitate RNA, and then add RNase free water to dissolve it completely to obtain RNA solution and store it in refrigerator at −80°C. The reverse transcription reaction was carried out by transcription kit (Takara RR036B, Japan). The reverse-transcripted cDNA was stored in a refrigerator at −20°C. According to the instructions of real-time fluorescent quantitative PCR kit (TaKaRa RR086B, Japan), the cDNA of each sample was amplified. The CT value was read from the computer (ABI StepOnePlus Real-Time PCR System). After the reaction, CT value was used as the result for relative quantitative analysis. The primer sequences were as follows: miR-21: forward primer, 5′-TTTCCCGAACCACCCTATCC-3′, reverse primer, 5′-CAGACTCTGCATGTTCCAGC-3′); caspase-3 mRNA: forward primer, 5′-GCTGGACTGCGGTATTGAGACA-3′, reverse primer, 5′- TGAACCATGACCCGTCCCTTGA-3′); caspase-9 mRNA: forward primer, 5′- CTTCACGCGCGACATGATC-3′, reverse primer, 5′-AGAGCTGGATTGTGGAAGGT-3′. The comparative Ct (2^−△△Ct^) method was used to analyze the relative expression of mRNAs.

### 2.6. Western Blot Analysis

The lymphocytes collected previously were lysed with lysis buffer, and the Bradford method was used to quantify the protein. The protein was electrophoretic by sodium dodecyl sulfate polyacrylamide gel electrophoresis (SDS PAGE). The protein was transferred to the polyvinylidene fluoride membrane by electrospinning. Antibody was used to incubate overnight. We coupled antibody with horseradish peroxidase: Primary antibodies were caspase-3 (Abcam, ab184787, UK), dilution ratio: 1 : 2000, and caspase-9 (Abcam, ab184786, UK), dilution ratio: 1 : 1000. Secondary antibody was goat anti-rabbit IgG HRP (kgaa35, Jiangsu Kaiji Biotechnology Co., Ltd., China). Bio-Rad imaging was analyzed by quantity one software.

### 2.7. IL-17 and IL-10 Were Tested by ELISA

IL-17 and IL-10 in serum and cell culture medium were determined by using rat ELISA Kit (China Kaiji Biotechnology Co., Ltd, KGERC170-1, KGERC004-1), and the absorbance (OD value) of the above cytokines was measured by using microplate reader (MD SpectraMax M3, USA), and the corresponding concentrations were calculated.

### 2.8. Apoptosis of Lymphocytes Was Detected by TUNEL

After the preparation of lymphocyte samples, they were immersed in paraformaldehyde solution overnight to improve the tissue permeability, and the samples were treated with methanol solution to inactivate enzymes. During TUNEL reaction, the samples were labeled with streptavidin TRITC working solution, and the lymphocyte samples were restained with DAPI working solution. After several times of washing with deionized water, the samples were observed with fluorescence microscope.

### 2.9. Detection of Caspase-3 and Caspase-9 Protein by Immunofluorescence

The separated lymphocyte was fixed with paraformaldehyde, inactivated with methanol solution, blocked with goat serum, incubated with primary antibody, and then incubated with FITC secondary antibody. DAPI staining solution was used to restain the cells, the caspase-3 and caspase-9 proteins were observed under fluorescence microscope, and photos were taken for preservation.

### 2.10. Statistical Analysis

The data shown represent the mean ± standard; *t*-test or ANOVA was used in our research. Statistical significance was expressed by *p* value (^*∗*^*p* < 0.05 and ^*∗∗*^*p* < 0.01).

## 3. Results

### 3.1. Changes of CD4+T, Th17, Treg Cells, and Inflammatory Factors

**Figure 1:**
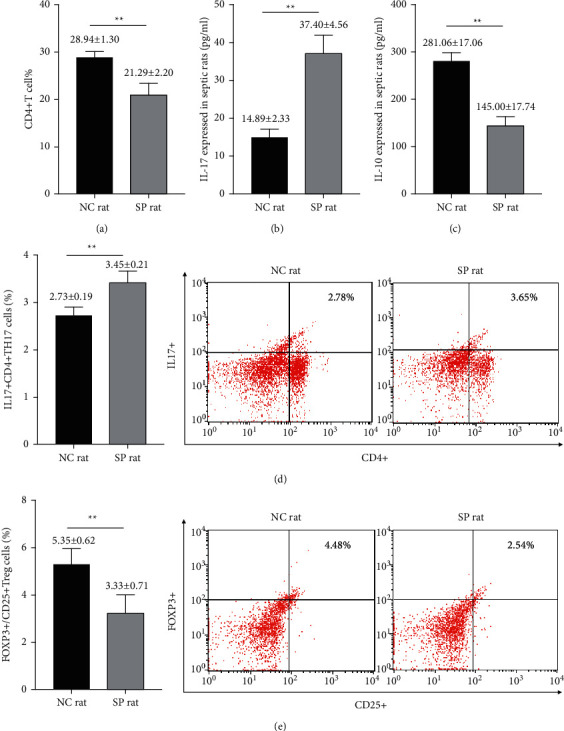
The proportions of lymphocytes and inflammatory factors were changed in peripheral blood of septic rats. (a) The ratios of CD4+ T cells in peripheral blood in eight-paired NC rat group and SP rat group were determined by flow cytometry [*t* value = 14.213, *p* < 0.01]. ((b) and (c)) The levels of IL-17 and IL-10 in peripheral blood in eight-paired NC rat group and SP rat group were determined by ELISA [*t* value (IL-17) = 16.892, *p* < 0.01; *t* value (IL-10) = 16.312, *p* < 0.01]. (d) The ratio of Th17 cells in peripheral blood in eight-paired NC rat group and SP rat group were determined by flow cytometry [*t* value = 5.687, *p* < 0.01]. (e) The ratios of Treg cells in peripheral blood in eight-paired NC rat group and SP rat group were determined by flow cytometry [*t* value = 5.482, *p* < 0.01]. NC group: normal control group; SP group: sepsis group. ^*∗∗*^*p* < 0.01.

We firstly found that the ratio of CD4+ T lymphocytes of septic rats was decreased when compared with the NC group [*t* value = 14.213, *p* < 0.01] (Figure 1(a)). Compared with the NC group, the ratio of Th17 of septic rats increased, while the ratio of Treg decreased [*t* value = 5.687, *p* < 0.01; *t* value = 5.482, *p* < 0.01] (Figures 1(d) and 1(e)). Compared with the NC group, IL-17 in the peripheral blood of septic rats increased significantly, while the level of IL-10 decreased significantly [*t* value (IL-17) = 16.892, *p* < 0.01; *t* value (IL-10) = 16.312, *p* < 0.01] (Figures 1(b) and 1(c)).

### 3.2. Changes of Apoptosis of Lymphocytes

**Figure 2:**
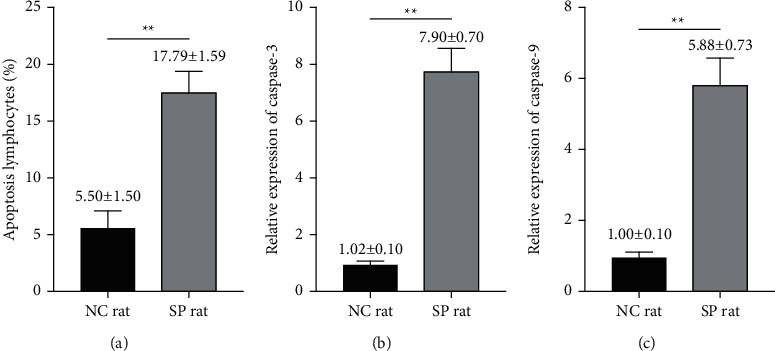
Indicators related to apoptosis were changed in lymphocytes of septic rats. (a) The percentages of apoptotic lymphocytes in eight-paired NC rat group and SP rat group were determined by flow cytometry [*t* value = 16.387, *p* < 0.01]. ((b) and (c)) The relative expressions of caspase-3 and caspase-9 mRNA in eight-paired NC rat group and SP rat group were determined by real-time quantitative PCR [*t* value (caspase-3) = 26.246, *p* < 0.01; *t* value (caspase-9) = 18.489, *p* < 0.01]. NC group: normal control group; SP group: sepsis group. ^*∗∗*^*p* < 0.01.

As reported, apoptosis is related to the differentiation and function of lymphocytes [9, 10]. So we detected apoptosis of lymphocytes by flow cytometry. We found that the apoptosis rate of lymphocytes of septic rats increased [*t* value = 16.387, *p* < 0.01] (Figure 2(a)). It is well known that the caspase family is responsible for selectively cleaving certain proteins, resulting in apoptosis. We detected the levels of caspase-3/9 mRNA by PCR. Compared with the NC group, the expression of caspase-3/9 mRNA in lymphocytes of septic rats increased significantly [*t* value (caspase-3) = 26.246, *p* < 0.01; *t* value (caspase-9) = 18.489, *p* < 0.01] (Figures 2(b) and 2(c)).

### 3.3. Changes of miR-21 in Lymphocytes

**Figure 3:**
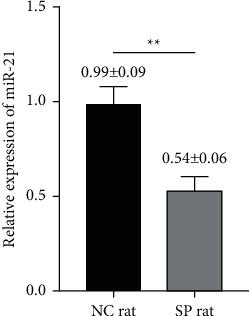
The relative expression of miR-21 was changed in lymphocytes of septic rats. The relative expressions of miR-21 in eight-paired NC rat group and SP rat group were determined by real-time quantitative PCR [*t* value = 23.037, *p* < 0.01]. NC group: normal control group; SP group: sepsis group. ^*∗∗*^*p* < 0.01.

As reported, miR-21 is closely related to apoptosis in medical fields [19–22]. We detected the expression of miR-21 by PCR. We found that the expression of miR-21 in lymphocytes of septic rats was significantly decreased [*t* value = 23.037, *p* < 0.01] (Figure 3).

### 3.4. Observation of Lymphocytes Transfected with miR-21

**Figure 4:**
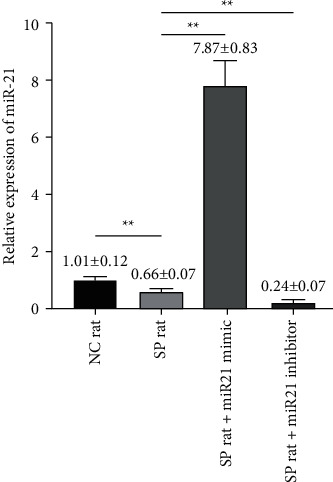
miR-21 activators and inhibitors were transfected into lymphocytes of septic rats. The relative expressions of miR-21 in eight-paired NC rat group, SP rat group, SP rat + miR21 mimic group, and SP rat + miR21 inhibitor group were determined by real-time quantitative PCR [*t* value (NC versus SP) = 5.436, *p* < 0.01; *t* value (miR-21 mimic versus SP) = 23.988, *p* < 0.01; *t* value (miR-21 inhibitor versus SP) = 18.393, *p* < 0.01]. NC group: normal control group; SP group: sepsis group. SP rat + miR21 mimic group: sepsis group with miR-21 activator transfected into lymphocytes; SP rat + miR21 inhibitor group: sepsis group with miR-21 inhibitor transfected into lymphocytes. ^*∗∗*^*p* < 0.01.

To observe whether miR-21 can regulate the differentiation and function of lymphocytes, we firstly need to establish a successful model of miR-21 transfection into lymphocytes in sepsis. In this study, we used miR-21 mimic and inhibitor for transfection into lymphocytes in sepsis. Compared with NC group and sepsis group, the expression of miR-21 in sepsis + miR-21 mimic group was increased, while the relative expression of miR-21 in sepsis + miR-21 inhibitor group was significantly reduced [*t* value (NC versus SP) = 5.436, *p* < 0.01; *t* value (miR-21 mimic versus SP) = 23.988, *p* < 0.01; *t* value (miR-21 inhibitor versus SP) = 18.393, *p* < 0.01] (Figure 4).

### 3.5. miR-21 Regulated the Differentiation and Function of Th17 and Treg Cells

**Figure 5:**
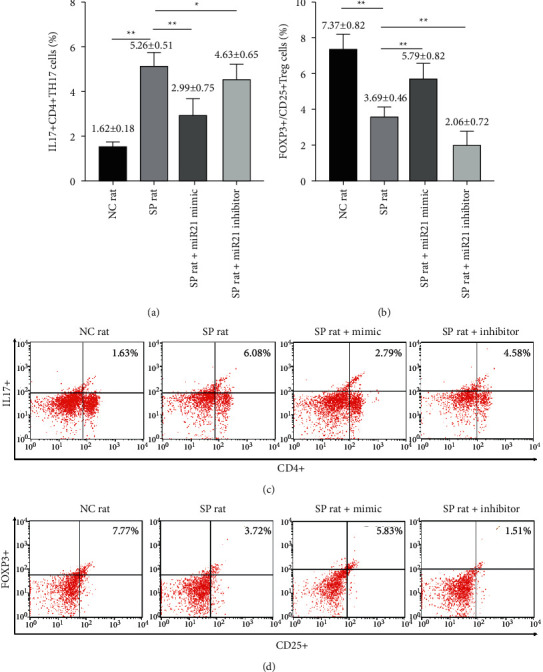
The ratios of Th17 and Treg cells in peripheral blood were changed after transfection of miR-21 activator or inhibitor into lymphocytes of septic rats. ((a) and (c)) The ratios of Th17 cells in peripheral blood in eight-paired NC rat group, SP rat group, SP rat + miR21 mimic group, and SP rat + miR21 inhibitor group were determined by flow cytometry [*t* value (NC versus SP) = 18.445, *p* < 0.01; *t* value (miR-21 mimic versus SP) = 6.362, *p* < 0.01; *t* value (miR-21 inhibitor versus SP) = 2.477, *p* < 0.05]. ((b) and (d)) The ratios of Treg cells in peripheral blood in eight-paired NC rat group, SP rat group, SP rat + miR21 mimic group, and SP rat + miR21 inhibitor group were determined by flow cytometry [*t* value (NC versus SP) = 9.836, *p* < 0.01; *t* value (miR-21 mimic versus SP) = 5.991, *p* < 0.01; *t* value (miR-21 inhibitor versus SP) = 5.180, *p* < 0.01]. NC group: normal control group; SP group: sepsis group; SP rat + miR21 mimic group: sepsis group with miR-21 activator transfected into lymphocytes; SP rat + miR21 inhibitor group: sepsis group with miR-21 inhibitor transfected into lymphocytes. ^*∗∗*^*p* < 0.01.

To observe the effect of miR-21 on lymphocytes in sepsis, we firstly enhanced or decreased the expression of miR-21 by lentivirus transfection. Next, we counted the Th17 and Treg cells ratio by flow cytometry again. The proportion of Th17 cells was significantly reduced, while the proportion of Treg cells was significantly raised in sepsis + miR-21 mimic group. There was no difference in the ratio of Th17 cells, while the ratio of Treg cells was significantly reduced in sepsis + miR-21 inhibitor group [Th17: *t* value (NC versus SP) = 18.445, *p* < 0.01; *t* value (Th17: miR-21 mimic versus SP) = 6.362, *p* < 0.01; *t* value (Th17: miR-21 inhibitor versus SP) = 2.477, *p* < 0.05; *t* value (Treg: NC versus SP) = 9.836, *p* < 0.01; *t* value (Treg: miR-21 mimic versus SP) = 5.991, *p* < 0.01; *t* value (Treg: miR-21 inhibitor versus SP) = 5.180, *p* < 0.01] (Figures 5(a)–5(d)).

### 3.6. Changes of Apoptosis after Intervention of miR-21 in Lymphocytes

**Figure 6:**
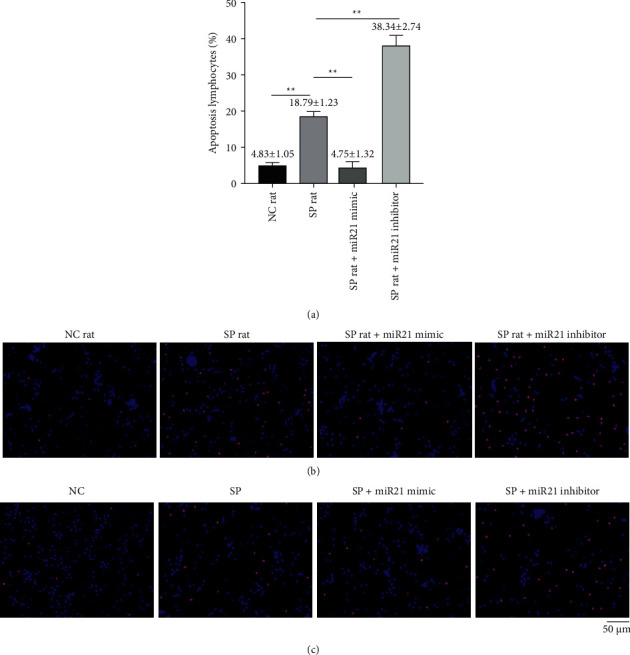
The percentages of apoptotic lymphocytes in peripheral blood were changed after transfection of miR-21 activator or inhibitor into lymphocytes of septic rats. (a) The percentages of apoptotic lymphocytes in eight-paired NC rat group, SP rat group, SP rat + miR21 mimic group, and SP rat + miR21 inhibitor group were determined by flow cytometry [*t* value (NC versus SP) = 23.026, *p* < 0.01; *t* value (miR-21 mimic versus SP) = 17.872, *p* < 0.01; *t* value (miR-21 inhibitor versus SP = 23.921), *p* < 0.05]. (b) In in vivo experiments, apoptotic lymphocytes were detected by immunofluorescence microscope in in eight-paired NC rat group, SP rat group, SP rat + miR21 mimic group, and SP rat + miR21 inhibitor group. (c) In in vitro culture, apoptotic lymphocytes were detected by immunofluorescence microscope in in eight-paired NC rat group, SP rat group, SP rat + miR21 mimic group, and SP rat + miR21 inhibitor group. NC group: normal control group; SP group: sepsis group. SP rat + miR21 mimic group: sepsis group with miR-21 activator transfected into lymphocytes; SP rat + miR21 inhibitor group: sepsis group with miR-21 inhibitor transfected into lymphocytes. ^*∗∗*^*p* < 0.01.

After miR-21 was overexpressed or inhibited, we observed apoptosis of lymphocyte of septic rats in vivo and in vitro using fluorescence microscopy. The apoptosis rate of lymphocytes in sepsis + miR-21 mimic group was significantly reduced, while that in sepsis + miR-21 inhibitor group was significantly raised [*t* value (NC versus SP) = 23.026, *p* < 0.01; *t* value (miR-21 mimic versus SP) = 17.872, *p* < 0.01; *t* value (miR-21 inhibitor versus SP = 23.921), *p* < 0.05] (Figures 6(a)–6(c)).

### 3.7. Changes of Caspase-3/9 after Intervention in Lymphocytes

**Figure 7:**
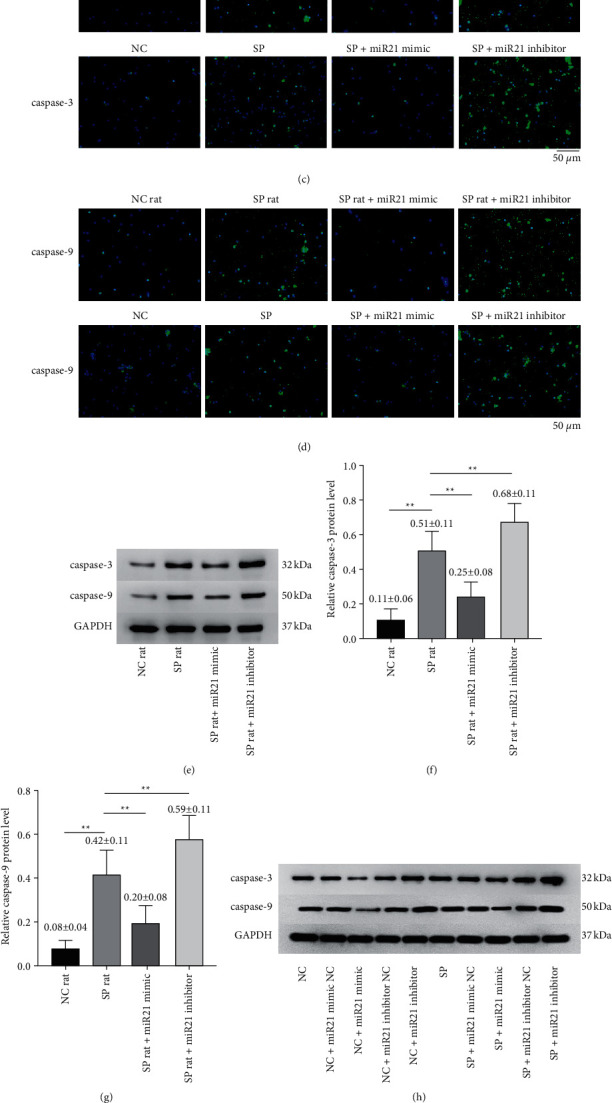
The levels of caspase-3 and caspase-9 in lymphocytes were changed after transfection of miR-21 activator or inhibitor into lymphocytes of septic rats. ((a) and (b)) The relative expressions of caspase-3 and caspase-9 mRNA in eight-paired NC rat group, SP rat group, SP rat + miR21 mimic group, and SP rat + miR21 inhibitor group were determined by real-time quantitative PCR [*t* value (caspase-3: NC versus SP) = 25.960, *p* < 0.01; *t* value (caspase-3: miR-21 mimic versus SP) = 10.874, *p* < 0.01; *t* value (caspase-3: miR-21 inhibitor versus SP) = 5.934, *p* < 0.01; *t* value (caspase-9: NC versus SP) = 13.300, *p* < 0.01; *t* value (caspase-9: miR-21 mimic versus SP) = 3.711, *p* < 0.01; *t* value (caspase-9: miR-21 inhibitor versus SP) = 12.930, *p* < 0.01]. ((c) and (d)) The expressions of caspase-3 and caspase-9 protein in eight-paired NC rat group, SP rat group, SP rat + miR21 mimic group, and SP rat + miR21 inhibitor group were determined by immunofluorescence microscope. ((e)–(g)) In in vivo experiments, the protein levels of caspase-3 and caspase-9 in eight-paired NC rat group, SP rat group, SP rat + miR21 mimic group, and SP rat + miR21 inhibitor group were determined by Western blot [*t* value (caspase-3: NC versus SP) = 10.643, *p* < 0.01; *t* value (caspase-3: miR-21 mimic versus SP) = 4.089, *p* < 0.01; *t* value (caspase-3: miR-21 inhibitor versus SP) = 4.545, *p* < 0.01; *t* value (caspase-9: NC versus SP) = 7.345, *p* < 0.01; *t* value (caspase-9: miR-21 mimic versus SP) = 4.664, *p* < 0.01; *t* value (caspase-9: miR-21 inhibitor versus SP) = 2.681, *p* < 0.05]. ((h)–(j)) In in vitro culture, the protein levels of caspase-3 and caspase-9 in eight-paired NC rat group, SP rat group, SP rat + miR21 mimic group, and SP rat + miR21 inhibitor group were determined by Western blot. NC group: normal control group; SP group: sepsis group; SP rat + miR21 mimic group: sepsis group with miR-21 activator transfected into lymphocytes; SP rat + miR21 inhibitor group: sepsis group with miR-21 inhibitor transfected into lymphocytes. ^*∗∗*^*p* < 0.01.

After miR-21 was overexpressed or inhibited, we observed changes of caspase-3/9 of septic rats in vivo and in vitro. We firstly observed that the fluorescence of caspase-3/9 decreased in sepsis + miR-21 mimic group, while the fluorescence of caspase-3/9 increased in sepsis + miR-21 inhibitor group [*t* value (caspase-3: NC versus SP) = 25.960, *p* < 0.01; *t* value (caspase-3: miR-21 mimic versus SP) = 10.874, *p* < 0.01; *t* value (caspase-3: miR-21 inhibitor versus SP) = 5.934, *p* < 0.01; *t* value (caspase-9: NC versus SP) = 13.300, *p* < 0.01; *t* value (caspase-9: miR-21 mimic versus SP) = 3.711, *p* < 0.01; *t* value (caspase-9: miR-21 inhibitor versus SP) = 12.930, *p* < 0.01] (Figures 7(a)–7(d)). Next we noticed that the expression of caspase-3/9 mRNA decreased significantly in sepsis + miR-21 mimic group, while the expression of caspase-3/9 mRNA increased significantly in sepsis + miR-21 inhibitor group (Figures 7(e) and 7(e)). Finally, we noticed that the levels of caspase-3/9 protein decreased in sepsis + miR-21 mimic group, while the levels of caspase-3/9 protein increased in sepsis + miR-21 inhibitor group [*t* value (caspase-3: NC versus SP) = 10.643, *p* < 0.01; *t* value (caspase-3: miR-21 mimic versus SP) = 4.089, *p* < 0.01; *t* value (caspase-3: miR-21 inhibitor versus SP) = 4.545, *p* < 0.01; *t* value (caspase-9: NC versus SP) = 7.345, *p* < 0.01; *t* value (caspase-9: miR-21 mimic versus SP) = 4.664, *p* < 0.01; *t* value (caspase-9: miR-21 inhibitor versus SP) = 2.681, *p* < 0.05] (Figures 7(g)–7(j)).

### 3.8. Changes of IL-17 and IL-10 after Intervention in Septic Rats

**Figure 8:**
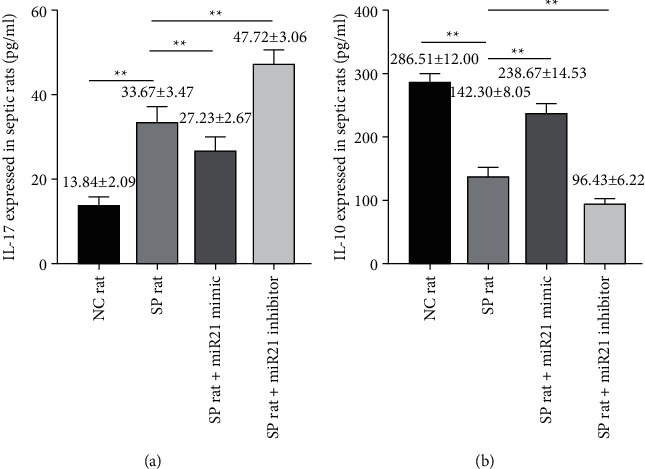
The levels of IL-17 and IL-10 in peripheral blood were changed after transfection of miR-21 activator or inhibitor into lymphocytes of septic rats. ((a) and (b)) The levels of IL-17 and IL-10 in peripheral blood in eight-paired NC rat group, SP rat group, SP rat + miR21 mimic group, and SP rat + miR21 inhibitor group were determined by ELISA [*t* value (IL-17: NC versus SP) = 13.660, *p* < 0.01; *t* value (IL-17: miR-21 mimic versus SP) = 5.096, *p* < 0.01; *t* value (IL-17: miR-21 inhibitor versus SP) = 8.638, *p* < 0.01; *t* value (IL-10: NC versus SP) = 29.703, *p* < 0.01; *t* value (IL-10: miR-21 mimic versus SP) = 18.042, *p* < 0.01; *t* value (IL-10: miR-21 inhibitor versus SP) = 14.523, *p* < 0.05]. NC group: normal control group; SP group: sepsis group; SP rat + miR21 mimic group: sepsis group with miR-21 activator transfected into lymphocytes; SP rat + miR21 inhibitor group: sepsis group with miR-21 inhibitor transfected into lymphocytes. ^*∗∗*^*p* < 0.01.

IL-17 decreased significantly in sepsis + miR-21 mimic group, while it increased significantly in sepsis + miR-21 inhibitor group [*t* value (IL-17: NC versus SP) = 13.660, *p* < 0.01; *t* value (IL-17: miR-21 mimic versus SP) = 5.096, *p* < 0.01; *t* value (IL-17: miR-21 inhibitor versus SP) = 8.638, *p* < 0.01; ] (Figure 8(a)); meanwhile, IL-10 increased significantly in sepsis + miR-21 mimic group, but it decreased significantly in sepsis + miR-21 inhibitor group [*t* value (IL-10: NC versus SP) = 29.703, *p* < 0.01; *t* value (IL-10: miR-21 mimic versus SP) = 18.042, *p* < 0.01; *t* (IL-10: NC versus SP) = 29.703, *p* < 0.01; *t* value (IL-10: miR-21 mimic versus SP) = 18.042, *p* < 0.01; *t* value (IL-10: miR-21 inhibitor versus SP) = 14.523, *p* < 0.05] (Figure 8(b)).

## 4. Discussion

Sepsis is a common disease in the ICU, with the characteristics of high mortality [23]. “The early inflammatory storm” theory cannot fully explain the whole pathophysiological process of sepsis, and various anti-inflammatory treatments alone can not improve its prognosis, so “immune paralysis” theory has gradually been widely valued by scholars [24]. Some scholars have proposed that apoptosis of immune cells induced by sepsis is one of the pathophysiological mechanisms of immunosuppression [2]. A study showed that T cell response was significantly reduced in patients with posttraumatic sepsis, in which Treg was significantly increased and Th17 was significantly reduced [10]. This result showed that the imbalance of Th17/Treg cells led to the disorder of inflammatory response and finally led to the occurrence of immunosuppressive state in sepsis. One basic experimental study also found that the level of Treg cells in the serum of septic mice increased, and the corresponding inflammatory factors secreted were significantly excessive. It was further found that knockout of TLR4 could reduce Treg activity and restore the balance of proinflammatory cytokines to improve immune paralysis [25]. Therefore, one of the recent studies about the treatment of sepsis mainly focuses on how to regulate the immune balance, that is, the so-called “Immunomodulatory Therapy” [26]. For example, Zou found that miR-126 could control the inflammatory response in peripheral blood by regulating the balance of Th17/Treg cells. In our study, in early stage of sepsis, Th17 cells increased significantly in the peripheral blood of rats, while Treg cells decreased significantly. The imbalance of Th17/Treg ratio is consistent with Zou's research [27]. We also found that IL-17 increased, while IL-10 decreased, indicating that this study is in line with “excessive inflammatory response” in early stage of sepsis.

One of the early characteristics of sepsis is that a large amount of lymphocyte apoptosis leads to decline in the number of lymphocytes [12, 28–31]. This study confirmed the same conclusion that the count of CD4+ T lymphocytes of septic rats reduced significantly. Similarly, clinical studies have found that the apoptosis of lymphocytes with sepsis patients increased significantly, while the lymphocyte count decreased significantly [32]. In order to find out the cause of the decline in the number of T lymphocytes, we found that the apoptosis rate of T lymphocytes in septic rats increased significantly. It is well known that the most important pathway related to apoptosis is caspase signaling pathway. Caspase family belongs to cysteine protease, which is responsible for selectively cleaving some proteins, resulting in apoptosis. Caspase-3 can directly degrade the structural and functional proteins in cells, thus causing apoptosis. After stimulation, caspase-9 can be activated by self-cutting and then cause caspase cascade reaction. In this study, we found that numerous apoptotic lymphocytes appeared, and caspase-3 and caspase-9 significantly increased, indicating that caspase pathway is involved in apoptosis of lymphocytes. Most scholars believe that apoptosis of lymphocyte is one of the causes of “immune paralysis” in sepsis, but some scholars have questioned this view [33–37]. In the early stage of sepsis, we found that inflammatory factors in peripheral blood were released in large quantities and apoptosis of lymphocytes appeared. Combined with the pathophysiological process of sepsis, the body was in the stage of “excessive inflammatory response” in the early stage. We also confirmed that the release level of IL-17 increased significantly but that of IL-10 decreased significantly, which was related to the imbalance of Th17/Treg ratio. This shows that the proportion of Th17/Treg cells is changed by apoptosis of lymphocyte in early sepsis, which makes the body in the stage of “excessive inflammatory response.” Similarly, in late sepsis, we guess that the proportion of Th17/Treg was affected by apoptosis of lymphocyte, which makes the body in the stage of “immune paralysis.” This may be the reason why simple anti-inflammatory treatment in the early stage of sepsis has not achieved good curative effect. Through our study, we speculate that the proportion of Th17/Treg should be balanced by immunotherapy in the early or late stage of sepsis, so as to make the body in the stage of “controllable inflammatory response” in the early stage and avoid “immune paralysis” in the late stage.

Many research works have explored miR-21 involvement in many aspects of sepsis, such as inflammation and MODS (liver, kidney, and lung) [38–40]. Our study proved that the expression of miR-21 in lymphocytes of sepsis also changed significantly, so miR-21 may regulate the immune cells, thereby affecting the immune state. Clinical studies found that miR-21 was negatively correlated with inflammation and disease severity in sepsis [41]. Animal model validation also obtained the same results; that is, miR-21 reduces the production of proinflammatory factors through its downstream pathway and reduces the level of inflammatory response in sepsis, thereby reducing tissue damage and organ dysfunction [40]. In this study, we also found that the increase of miR-21 can lead to the change of inflammatory factors in peripheral blood of septic rats, such as IL-17 and IL-10. This study further found that miR-21 moderates the inflammatory response through affecting the differentiation of Th17/Treg; that is, the increase of miR-21 can reduce the count of Th17 cells but increase the count of Treg cells, resulting in different proportions of inflammatory factors secreted by them. Some articles have demonstrated that miR-21 participates in the process of apoptosis; for example, knockout of miR-21 gene can increase the apoptosis of renal tubular endothelial cells induced by ischemia-reperfusion [42]. With the increase of miR-21, the apoptosis of lymphocytes was significantly reduced, and caspase-3 and caspase-9 proteins were significantly decreased, and vice versa. These results indicate that miR-21 in lymphocytes of sepsis affects the differentiation of Th17/Treg cells through apoptosis pathway, thereby affecting inflammatory response and immune function.

In conclusion, in the early stage of sepsis, the body is in the stage of excessive inflammatory response, which may be caused by the imbalance of lymphocyte subsets (Th17/Treg cell ratio). The apoptosis process involved in caspase pathway changes the whole process of differentiation and function of Th17/Treg cells. Regulating apoptosis of lymphocyte to improve immune balance may be the key to improve the inflammatory response in the early stage of sepsis and may also be one of the means to prevent “immune paralysis” in the late stage of sepsis. Our study found that miR-21 exhibited a certain effect on differentiation and function of lymphocyte subsets in sepsis and can affect the inflammation and immune balance of sepsis. It can be used as one of the markers of sepsis and a potential target for sepsis treatment, However, in order to achieve the balance of Th17/Treg cells in sepsis, the opportunity of miR-21 activation or inhibition is unclear and needs to be confirmed by further research.

## Data Availability

The data used to support the findings of this study are included within the manuscript , and if needed more, can be asked to submit more by corresponding author.
